# Array-Patterned Anisotropic Conductive Films for High Precision Circuit Interconnection

**DOI:** 10.3390/ma18214927

**Published:** 2025-10-28

**Authors:** Changxiang Hao, Junde Chen, Yonghao Chen, Ge Cao, Xing Cheng, Yanqing Tian

**Affiliations:** 1 Department of Materials Science and Engineering, Southern University of Science and Technology, Shenzhen 518055, China; 12231017@mail.sustech.edu.cn (C.H.); 12132030@mail.sustech.edu.cn (Y.C.); 2 Institute of Corrosion Science and Technology, Guangzhou 510530, China; 3 School of Textile, Guangdong Polytechnic, Gaoming, Foshan 528000, China

**Keywords:** template with microcavities, well-aligned conducting particles in resin film, array-patterned anisotropic conductive films, high-precision circuit connection

## Abstract

Anisotropic conductive films (ACFs) are widely used for circuit interconnection due to their easy use, low temperature bonding, higher precision than soldering and eco-friendliness. However, current ACFs are generally prepared by randomly distributing conductive particles into suitable resins. The ACFs prepared by this approach have risks to result in shortcut when applied for high precision bonding (<100 μm). In order to alleviate this problem, we designed and prepared a new kind of ACFs with conducting particles well aligned in adhesive film, which is named as array-patterned ACFs (A-ACFs). A template with 12 μm periodic microcavities was prepared and used to load 5.4 μm silver-coated polystyrene particles. Through a series of process optimizations including particles-filling cycles and particles-transferring-pressure/temperature into the used polyurethane (PU) adhesive, well-aligned particles with a spacing of 6.6 μm in the PU film was obtained. Such prepared A-ACFs were used to bond two flexible printed circuits (FPC) not only with a spacing of 200 μm (FPC-200) but also with 40 μm (FPC-40). The bonding conditions including temperature and pressure for the FPC-200 connections were investigated in detail. The connecting resistance, insulation resistance, peeling force, and the particles’ morphologies between the bonded FPCs were investigated. The reliability of the two bonded FPCs were tested under 85 °C and 85% relative humidity. Results showed that the new kinds of A-ACFs are suitable for achieving high precision circuits bonding and show better accuracy than those of traditional ACFs (T-ACFs). Thus, this study might have new insight for designing A-ACFs and great potential for applications in high-precision devices.

## 1. Introduction

Anisotropic conductive films (ACFs) are functional conductive adhesive materials that conduct along the vertical (*Z*-axis) direction while remaining insulating in the plane (*X*–*Y* axes) [1,2,3]. They typically comprise a curable resin matrix and micrometer-scale conductive particles, which in fine-pitch interconnection scenarios are commonly spherical with diameters of 3–5 μm. Owing to their capability for fine interconnects and reduced thermal load, ACFs have been widely used for heterogeneous interconnections in mobile phones, liquid-crystal displays (LCDs), televisions, LEDs, PDPs, and OLEDs—such as FOF, FOG, COG, COF, and COP configurations [4,5,6,7,8]—and their applications are extending to emerging areas including flexible wearable devices and micro-LEDs [9,10]. Compared with conventional soldering, ACFs can achieve high-density interconnections at pitches of several tens of micrometers and even around 10 μm, with processing temperatures typically in the range of 140–200 °C, significantly lower than the >250 °C required for soldering [11,12,13,14,15,16,17,18,19,20]. These attributes confer distinct advantages for packaging heat-sensitive materials and for high-density assembly.

To support the high-density assembly and packaging of heat-sensitive materials, several studies have demonstrated the advantages and developments of ACFs. Cao et al. (2005) formulated and characterized anisotropic conductive adhesive pastes for microelectronics packaging applications, showing enhanced performance by tailoring the resin matrix and particle size for fine-pitch interconnections [21]. Lin et al. (2011) proposed an automatic inspection method for evaluating fracture conditions of ACFs during the TFT-LCD assembly process, providing insights into how particle distribution affects the reliability and integrity of ACF-based interconnections in large-scale production [22]. Shin et al. (2017) explored the encapsulation of imidazole with synthesized copolymers, enabling the latent curing of epoxy resin, which contributes to the improved thermal stability and bonding reliability of ACFs during the curing process [23].

Furthermore, Zhang et al. (2017) optimized bonding parameters for flex-on-board assemblies, focusing on Sn58Bi solder joint morphology when using ACFs, and demonstrated how improved bonding protocols enhance the robustness of ACF interconnections in flexible devices [24]. Ham et al. (2010) enhanced the thermal and mechanical properties of ACFs by microencapsulating imidazole curing agents, showing improvements in high-temperature bonding stability [25]. Suppiah et al. (2017) reviewed adhesive bonding in semiconductor interconnections, discussing the role of ACFs in achieving scalable and reliable interconnections for advanced microelectronics [26]. Lee et al. (2020) used nanoscale dewetting for direct interconnections in microelectronics, contributing to deterministic assembly methods that can be applied for high-precision ACF transfer printing [27].

Despite the maturity of the industry-standard “random mixing–coating” fabrication route, traditional ACFs (T-ACFs) face predictable reliability bottlenecks because conductive particles are randomly distributed within the film: local agglomeration and uneven distributions can trigger short circuits between adjacent electrodes, thereby constraining deployment under higher densities and narrower process windows [28,29,30]. To suppress such failure modes, array-patterned ACFs (A-ACFs) arrange conductive particles periodically and regularly within the film, reducing design-level uncertainty arising from electrical shorts and statistical variation [31]. Prior efforts include block-copolymer templating to perforate a film and fill it with ~20 μm particles for flexible devices; however, particle size and geometric limitations hinder suitability for finer-pitch interconnections [32]. Our group previously proposed a silicon-template-based fill-then-transfer process to fabricate A-ACFs, laying the groundwork for arraying, but systematic optimization of particle-transfer parameters, bonding window, and the microstructures within the bonded region remained insufficient [33]. Consequently, developing a methodology for arrayed ACFs that addresses the full “materials–structure–process–performance” chain for micro-pitch interconnects holds clear academic and engineering value.

Building on this, this work employs a polyurethane (PU) matrix with 5.4 μm conductive particles under mild conditions and proposes—then systematically optimizes—a fabrication and bonding strategy for A-ACFs aimed at high-density interconnections. Specifically, under low-temperature/low-pressure conditions of 120 °C and 3.76 kPa, we achieve ordered transfer of particles from a template into the PU adhesive film, obtaining a regular array with an inter-particle spacing of ~6.6 μm; subsequently, reliable electrical connections at a minimum pitch of 40 μm are realized at 160 °C and 2.01 MPa, with contact resistance as low as ~0.1 Ω over a 0.4 mm^2^ contact area and insulation resistance exceeding 200 MΩ. Importantly, the samples maintain stable conduction and insulation after 200 h of accelerated aging at 85 °C/85% RH, demonstrating strong environmental reliability and process robustness. Compared with existing A-ACFs and T-ACFs, this work achieves synergy across “small particle size–low-temperature/low-pressure transfer–regular array construction–low-temperature bonding window–long-term stability”: arrayed layouts suppress failure mechanisms associated with random distributions, while mild thermomechanical conditions broaden compatibility with heat-sensitive substrates and devices, thereby providing a replicable and scalable materials-and-process pathway for next-generation micro-pitch interconnections. Compared with other approaches, this work enables the bonded circuits to withstand stretching or bending [33], while achieving higher bonding precision [32].

## 2. Material and Methods

### 2.1. Chemicals and Materials

RZJ-304-50 photoresist was purchased from Ruihong Electronic Chemicals Co., Ltd. (Suzhou, China). Polyurethane 1624 (PU) was purchased from Shenzhen Jitian Chemical Co., Ltd. (Shenzhen, China). The glass transition temperature of PU after drying is 75 °C, with an initial viscosity of 3600 mPa·s. The conducting particles with 5.4 μm diameter of polystyrene covered with 190 nm silver was prepared according to our previous report [33], The volume resistivity of the particles is 2.6 × 10^−4^ Ω·cm (under a pressure of 10 MPa). FPC circuit (The volume resistivity is 1.9 × 10^−6^ Ω·cm.) with ten lines of 200 μm in spacing and pad (FPC-200) was purchased from Shenzhen Fujinglong Electronics Co., Ltd. (Shenzhen, China). FPC-40 (the volume resistivity is 1.72 × 10^−6^ Ω·cm) was purchased from Shenzhen Ruige Ruisi Technology Co., Ltd. (Shenzhen, China). The FPC-40 has two major lines, each with 15 branch lines. The 15 branch lines of the two major lines alternate adjacently but staggered (Figure S1). The spacing between each branch lines is 30 μm; the spacing between each pad is 40 μm. Rubber eraser was purchased from DELI Group Co., Ltd. (Ningbo, China). PET with 200 g release force was purchased from Shenzhen Zhenhua Adhesive Products Co., Ltd. (Shenzhen, China). Ethanol and acetone were provided by Bide Pharmaceutical Technology Co., Ltd. (Shenzhen, China). The high-precision micrometer (MDC-25MX) was purchased from Mitutoyo Corporation (Kawasaki, Japan).

### 2.2. Instrument

Hot press HK-3052 for bonding was purchased from Shenzhen Huake Lida Automation Equipment Co., Ltd. (Shenzhen, China); IKA RET basic station for heating template was purchased from IKA Instrument Equipment Co., Ltd. (Guangzhou, China); Instron 2367 tensile machine was ordered from Instron Corporation (Norwood, MA, USA), which is for testing peel strength performed at an angle of 180° at 5 mm/min according to the procedure of ASTM D3330; UTR2811D-II ohmmeter was purchased from Shenzhen Weifeng Instrument Co., Ltd. (Shenzhen, China). Inverted metallurgical microscope MJ42 was purchased from Mingmei Optoelectronics Technology Co., Ltd. (Guangzhou, China). Scanning electron microscope (Hitachi, SU8230, Hitachi City, Japan) was used for morphology observation, operated at 10 kV under vacuum.

### 2.3. Methods

#### 2.3.1. Preparation of Silicon Template with Microcavities

The preparation of the templates is according to our previous report [33]. The resulting silicon possesses an array of microcavities with a diameter of 6 μm, depth of 4 or 5 μm depending on etching time, and a spacing of 6 μm among the microcavities.

#### 2.3.2. Preparation Process of A-ACFs

(1): Arranging the conducting particles into the microcavities on silicon wafer.

**Figure 1:**
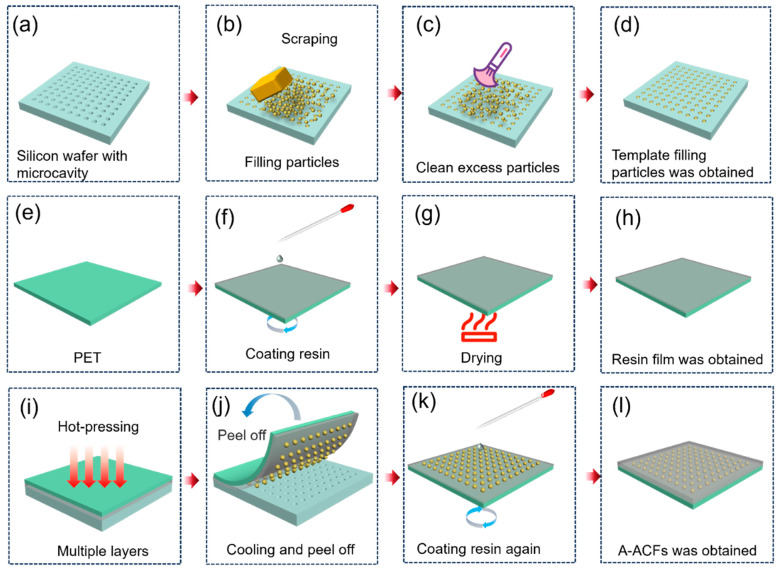
The preparation process of A-ACFs. (**a**) Blank template; (**b**) filling particles into the microcavity of the template; (**c**) cleaning excess particles on the template; (**d**) microcavity filled with particles; (**e**) blank polyethylene terephthalate (PET) film as a substrate of the A-ACFs; (**f**) spin coating polyurethane (PU) resin on PET; (**g**) drying the PU film; (**h**) PU film on the PET substrate (PFP); (**i**) laminating the PFP onto the particle-filled template with heat; (**j**) peeling off the particles-containing PFP; (**k**) spin coating another layer of PU on the particle-filled adhesive film; (**l**) PET carrier with A-ACFs.

The silicon template with microcavities (Figure 1a) was immersed in a 1:1 mixture of alcohol and acetone for cleaning under ultrasonic. The wafer was then dried in a 70 °C oven. A small amount of conducting particles was placed on the dried wafer template. A rubber was used to move the conducting particles into the microcavity (Figure 1b). Excess conducting particles were recovered (Figure 1c). The silicon wafer containing the conducting particles (Figure 1d) was placed on a hotplate for 2 min to enable the template to reach a predetermined temperature of 120 °C for the next step use.

(2): Transferring the conducting particles into the PU film.

PU was chosen as the adhesive. PET (Figure 1e) was used as the substrate for PU. To facilitate later separation of the PU adhesive film from PET substrate when use, PET with a few nanometer silicon oil, possessing the degree of release of 200 g was used. A 53 wt.% of PU in water was spin coated on the PET surface at 450 rpm for 10 s and 5000 rpm for 20 s. The film was then dried in a 70 °C oven for 30 min (Figure 1f–h). The film thickness is controlled by spin coating and drying times (Table S1). The PU coated PET film was laminated onto the wafer at 120 °C by applying a press of 3.76 KPa for 10 s and then the film was cooled to room temperature in air within 30 s. The PET film with PU was separated perpendicularly from the silicon wafer to finish the transferring of the microspheres from silicon wafer into the PU film. The PU adhesive with conducting particles on PET substrate (Figure 1i) was then placed on a spin coater with the conducting particles facing up. Another layer of PU was spin coated over the conducting particles (Figure 1j,k). The obtained film was dried in a 70 °C oven for 30 min to result in the ready-to-use A-ACFs (Figure 1l). The PET film will be removed before the use of A-ACFs for bonding.

#### 2.3.3. Preparation of T-ACFs

A 53 wt.% PU in water was spin coated on the PET surface at 450 rpm for 10 s and 5000 rpm for 20 s. The PU was dried in a 70 °C oven for 30 min to obtain the blank PU film. The film thickness is controlled by spin coating and drying times (Table S1). A total of 18.8 g of the 53 wt.% PU was placed in a beaker. Then, 0.05 g of the conducting particles was added into the beaker with PU. This mixture was stirred by magnetic stirring for 8 h to disperse the conducting particles in the PU adhesives. The PU resin containing particles was then spin coated on the surface of the previously prepared blank PU film at 450 rpm for 10 s and 5000 rpm for 20 s. After the film was dried in a 70 °C oven for 30 min, T-ACFs were obtained, which contains 0.5 wt.% of the conducting particles in the PU film.

#### 2.3.4. Bonding of the FPCs

For achieving reasonable bonding, bonding pressure was set in a range of from 1.51 MPa to 2.77 MPa. The pressure below 1.51 MPa of hot press HK-3052 instrument cannot be operated; the pressure exceeding 2.77 MPa will damage the circuits. Bonding temperature in a range from 100 °C to 220 °C was selected, which is the conventional bonding temperature range applied in industry.

Two kinds of FPCs with different spacing of the electric circuits were used. The FPC-200 (Figure S2) is for the study of connecting resistance, insulation resistance and peel force; the FPC-40 with high circuit precision is for the test of whether the chosen ACFs can be successfully applied to high-precision devices.

Before the bonding, the PET substrate was removed from the A-ACFs. The PET-free A-ACFs were placed between two FPCs on a hot-stage of the bonding instrument (Hot press HK-3052) and aligned to make sure the circuit lines of the top FPC were at the same position of the bottom FPC, which was viewed under a Charge Coupled Device (CCD) on the hot press (Hot press HK-3052). The hot press head used in these experiments was 2 mm width. The circuits (FPC-200) connecting area is calculated to be 0.4 mm^2^ (0.2 mm * 2 mm). The aligned FPC-ACF-FPC samples were then hot-pressed under varying pressures and temperature settings to achieve the bonding of the FPCs using ACFs. The bonding time for all this study is set at 20 s. After the pressure was released the two FPCs were bonded together using the chosen ACFs.

#### 2.3.5. Electrical Resistance Measurement of the Bonded FPCs

Circuit resistances between FPCs were tested using an electronic bridge instrument UTR2811D-II ohmmeter. Figures S1 and S2 illustrate the resistance testing method for the bonded FPCs. The ACFs bonded circuits were placed on the platform. Using two probes of the UTR2811D-II ohmmeter, the conductive resistance at the two ends of the same circuit was measured, while the insulating resistance between adjacent circuits at the same side was measured. Environment effect at 85 °C and 85% relative humidity conditions for different periods of time on the electric resistance is also conducted.

#### 2.3.6. Peel Force Test

According to the ASTM D3330 method, the peel force testing method used in this study was conducted using the Instron 2367 material testing system at a speed of 5 mm/min. The two ends of the bonded circuit were clamped in the grips of the tensile machine. Data from each measuring condition was repeated for five times and the data was averaged.

#### 2.3.7. The Testing Method of Electrochemical Current Changes in the Flexible Circuit

As with the other mechanical tests [34],A self-made cyclic test equipment was used to test the flexible circuits. It enables reciprocating motion over a fixed distance. The working setup is shown in Figure S3. The probes of the electrochemical workstation were connected to the two ends of the flexible device, with the voltage of the electrochemical workstation kept constant at 1.5 V. The testing time was 14,000 s, bending radius is 0.5 cm, and twisting angle is 180°. One cycle test of the homemade cycling machine is 4 s. In these tests, 3500 cycles were studied. The percentage change in current was obtained by comparing the instantaneous current change with the initial current change (Figure S3). The electrochemical current change was monitored in real time during the application of cyclic stress. Based on the current versus time data, the impact of stretching, bending, and twisting cycles could be evaluated.

### 2.4. Characterization

**Figure 2:**
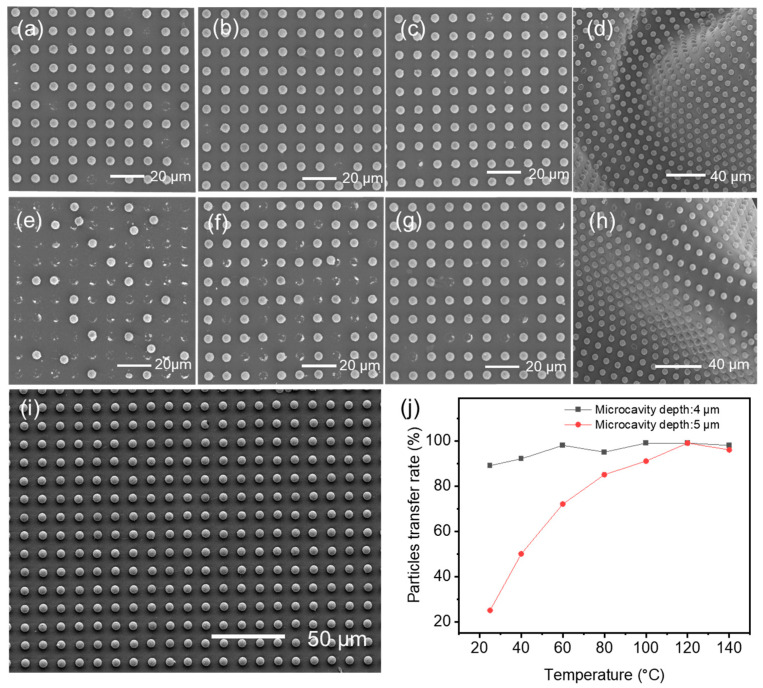
SEM images of transferred particles from the microcavity of the template to the PFP film under different parameters using the same pressure of 3.76 KPa and time of 10 s (**a**–**h**). (**a**) 4 μm depth operated at 25 °C; (**b**) 4 μm depth operated at 60 °C; (**c**) 4 μm depth operated at 100 °C; (**d**) 4 μm depth operated at 140 °C; (**e**) 5 μm depth operated at 25 °C; (**f**) 5 μm depth operated at 60 °C; (**g**) 5 μm depth operated at 100 °C; (**h**) 5 μm depth operated at 140 °C; (**i**) 4 μm microcavity depth operated at 120 °C; (**j**) Particle transfer efficiency varies with depth of microcavity and temperature.

**Figure 3:**
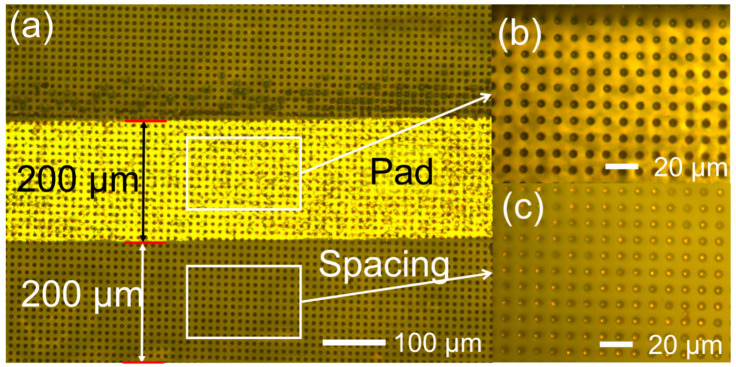
Images of metallographic microscopes. (**a**) A-ACFs attached on a FPC-200; (**b**) enlarged image at pad; (**c**) enlarged image at spacing.

Consistent with the preceding methods [35,36,37], morphology and particle arrangement were examined using an inverted metallurgical microscope (MJ42, Mingmei Optoelectronics, Kunshan, China) and a field-emission scanning electron microscope (SU8230, Hitachi, Tokyo, Japan) operated at 10 kV under high vacuum. Unless otherwise noted, imaging was performed on A-ACF after particle transfer and on bonded FPC cross-sections prepared from representative joints. The silicon templates with microcavities (6 μm diameter, 6 μm pitch, 4 or 5 μm depth) were also inspected to confirm cavity geometry prior to particle loading. For particle-array assessment, both plan-view optical images and SEM micrographs were collected across multiple fields along the web direction to qualitatively verify long-range order and to compare the influence of transfer temperature and cavity depth, in accordance with the datasets shown in Figure 2 and Figures S6–S9. The evolution of particle ordering during bonding (temperature/pressure windows) was evaluated from metallographic images of A-ACFs positioned on FPCs (Figure 3 and Figure S10). Post-bond cross-sectional observations were used to assess particle integrity (fracture vs. intact), metal-shell continuity, and contact conformality to both pads under different bonding conditions (Figure S11).

**Figure 4:**
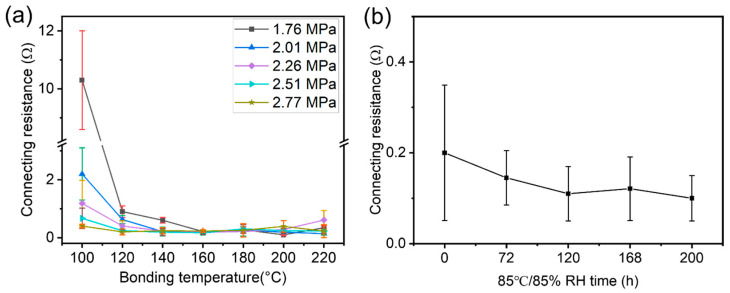
Connecting resistance influenced by bonging conditions and aging. (**a**) Connection resistance under various bonding pressures and temperatures; (**b**) shift in connecting resistance of the two boned FPC-200s by using A-ACFs during 85 °C/85% RH aging.

Electrical performance of bonded assemblies was characterized using a digital bridge ohmmeter (UTR2811D-II, Weifeng, Shenzhen, China). The connection (through-thickness) resistance was measured between the two pads of the same circuit line on the mated FPC pair, while insulation resistance was measured between adjacent, non-connected lines on the same side (measurement layouts in Figures S1 and S2). For FPC-200, bonding was performed across a matrix of temperatures (100–220 °C) and pressures (1.76–2.77 MPa), and the corresponding connection resistance map was compiled (Figure 4a). For FPC-40, the optimized bonding window (160 °C, 2.01 MPa) was used and both connection and insulation resistances were recorded (Table S2). Environmental stability was assessed by aging bonded samples at 85 °C and 85% relative humidity (RH); connection resistance drift was tracked over time (Figure 4b).

**Figure 5:**
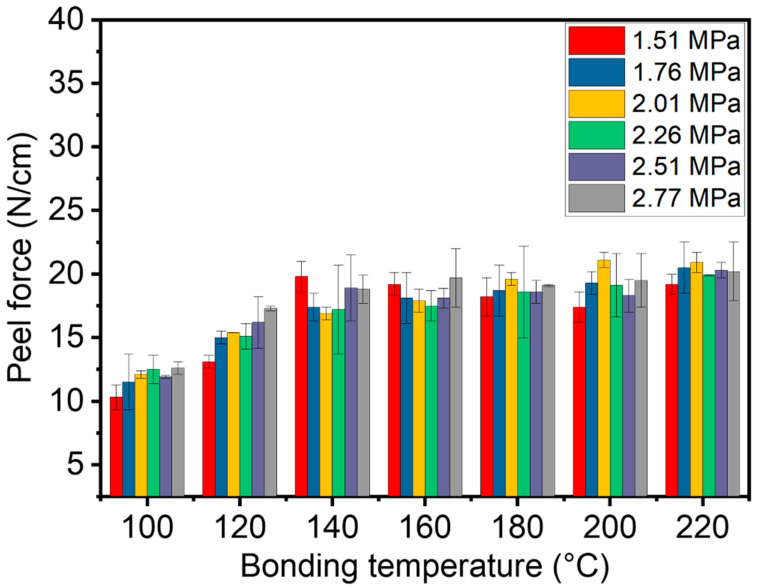
Effect of bonding pressures and temperatures on the peel force between the two connected FPC-200s using the A-ACFs.

Adhesion was quantified by 180° peel tests on FPC-200 joints using a universal testing machine (Instron 2367) at 5 mm min^−1^ following ASTM D3330. Each condition was measured five times and averaged to report the peel force (Figure 5). The temperature and pressure dependence of adhesion was analyzed to correlate resin flow/wetting with interfacial strength.

Flexibility and electromechanical stability were evaluated on bonded FPC-200 specimens using a custom cyclic bending/twisting setup (Figure S3). Samples were biased at a constant 1.5 V while monitoring current in real time over 14,000 s (≈3500 cycles; one cycle ≈4 s). The bending radius was 0.5 cm and the twisting angle was 180°. Percentage current change relative to the initial value was used as the stability metric to quantify the impact of cyclic mechanical deformation.

Collectively, this characterization framework—correlating microstructure (ordering, contact morphology), electrical metrics (connection/insulation resistance, aging drift), adhesion (peel force), and electromechanical robustness (cyclic bending/twisting)—supports the process–structure–property relationships established for the array-patterned ACFs and underpins the selection of the optimized bonding window (160 °C, 2.01 MPa).

## 3. Results and Discussion

### 3.1. Preparation of A-ACFs

#### 3.1.1. Design of the Template for the A-ACFs

Here, we designed a silicon template with microcavity to arrange the conductive particles. The preparation procedure for the template was given in Supporting Information (Figure S2). The template possesses circular microcavity with diameter of 6 μm, a spacing of 6 μm, and depth of 4 μm or 5 μm. Figure 1 describes the detailed preparation process of these A-ACFs.

#### 3.1.2. Filling Conductive Particles into Microcavity

**Figure 6:**
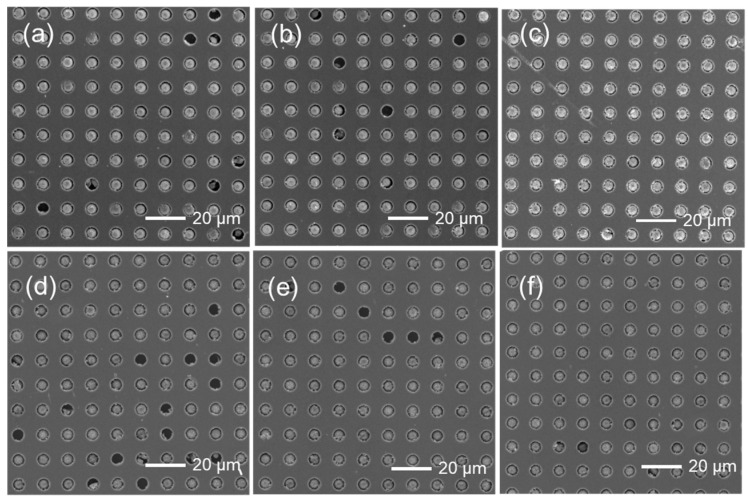
SEM of particle filling in the microcavity at different repeating time. (**a**) Once for the 4 m depth microcavity; (**b**) three times for the 4 m depth microcavity; (**c**) five times for the 4 m depth microcavity; (**d**) once for the 5 m depth microcavity; (**e**) three times for the 5 m depth microcavity; (**f**) five times for the 5 m depth microcavity.

**Figure 7:**
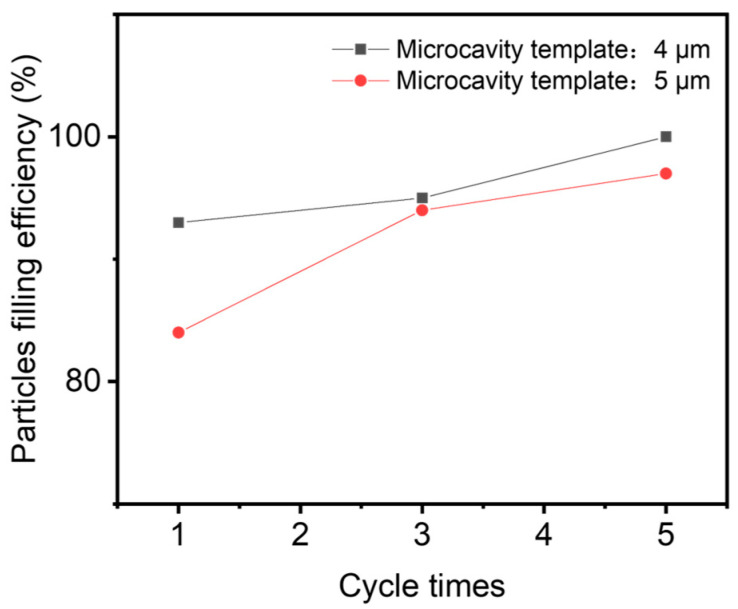
The effect of repeating cycle numbers on particle filling efficiency.

Figure 1a–d demonstrated the process of transferring particles into the template. Excess conductive particles were collected for reuse. For comparison, we prepared two kinds of templates with same diameters and spacing, but with different depths. One is at 4 μm depth; another one is at 5 μm depth. To ensure that each microcavity is filled with one particle, the rubber scraping was repeated three to five times (Figure 6). Figure 7 shows the effect of rubbing cycles on the particle filling percentages. Examination of the microcavities revealed complete filling after five iterative filling cycles (Figure 6). Research has found that there is not much difference in the filling effect between microcavities with 4 μm and 5 μm.

Based on previous studies on particle friction [38], the filling of particles into the template must satisfy the following conditions: first, the adhesion energy between particles and the upper substrate (Ep-s) should be greater than both the attractive energy between particles (Ep-p) and the adhesion energy between particles and the lower substrate (Ep-s1), so as to ensure that the conductive particles can be adsorbed by the upper substrate. The external shear force applied to the aggregated conductive particles must be transmitted to all conductive particles. Since the conductive particles filled in the grooves are not in contact with the upper substrate, they remain in the grooves by virtue of the blocking effect of the groove walls; the principle is illustrated in Figure S4.

#### 3.1.3. The Temperature Effect on Particle Transfer Efficiency from the Template to PU Adhesives

PU was used as the adhesive, which was dripped onto the PET surface (Figure 1f). The thickness of the PU adhesive film can be controlled basing on the number of spin coating and drying cycles (Table S1), our results showed that the thickness (3–34 μm) of PU has no effect on the efficiency of particle transferring from the template to the PU film. For this study, 10 μm PU film was chosen for the following experiments. The particle side of the template was laminated with PU film on the PET substrate (PFP) at various temperature and pressure (Figure 1g–i). After the films were cooled to room temperature, the conducting particles-containing PFP was peeled off from the template (Figure 1j).

Figure 2 showed the particles’ distribution on the PFPs, prepared at different temperatures, using the two microcavity at different depths, respectively. More SEM results at various temperatures were given in Figures S6 and S7. Generally, particle transfer efficiency is higher at a relative higher temperature. However, when temperature is set at 140 °C, significant deformation of the PU film was observed (Figure S6). Beautiful particles’ distributions in the PFP films were observed when the transfer temperature was set at 120 °C (Figure 2j). The depths of the microcavity from the template also has some influences on the particle’s transfer efficiency. The transfer efficiency of 4 μm depth template is higher than that of the 5 μm depth template (Figure 2i), since the protrusion of the particles is helpful for the interactions among particles and PU adhesive. Other conditions like hot pressing time and pressure have relatively small effects on particle transfer efficiency at the same temperature (Figures S8 and S9). We prioritize a pressure of 3.76 kPa and a hot-pressing time of 10 s. Therefore, unless noted, the experimental operations for further study were performed using samples prepared by filling particles into the 4 μm depth microcavity for five cycles. Using the optimal transfer parameters, the exact yield rate of successfully transferred particles is over 80%.

The use of chosen PU as a kind of adhesive has certain advantages. Glass transition temperature (*T*_g_) of this PU is 75 °C, which is a mild temperature. When the temperature exceeds *T*_g_ of PU, the PU film becomes more adhesive, which facilitates transferring particles onto the PU film. After the conducting particles were transferred to the PU films, another layer of PU adhesive was spin coated on the microsphere side of the PFP film (Figure 1k) to ensure the particles were wholly embedded in the PU films to achieve A-ACFs (Figure 1l).

During this process, the attractive energy between the adhesive film and the conductive particles (Ep-s2) is required to be greater than the attractive energy between the conductive particles and the template (Ep-s1), as illustrated in Figure S5. Only when Ep-s2 > Ep-s1 can it be ensured that the conductive particles are transferred along with the adhesive film.

### 3.2. Influence of Bonding Conditions on Electrical Resistance of the Bonded FPCs

In this study, two FPCs with circuit line spacing of 200 μm (FPC-200) were connected by using A-ACFs to study the influence of bonding temperature and pressure on the bonded FPC’s electric resistance and interconnected strength and also demonstrate the A-ACFs’ practical application. To achieve the interconnections of the two FPCs, a hot-press machine applying suitable pressure and temperature was used for bonding, consistent with widely used device-assembly methods in biosensor systems [39,40]. Figure 3a shows the optical micrograph of A-ACFs attached on an FPC-200. Uniform conductive particles’ distribution and arrangement were observed at spacing (Figure 3b) and pad (Figure 3c). More temperature and pressure influence on the conductive particles’ arrangement of the A-ACFs on two bonded FPCs were given in Figure S10. As pressure and temperature increase, the particles become disordered. Temperature has a greater influence on particle distribution than pressure. Both the disorderly arrangement of particles and particle aggregations are detrimental to high precision bonding. Therefore, appropriate bonding parameters need to be considered. At temperatures below 160 °C, and pressures below 2.01 MPa, the particles remain in a well-ordered array.

As for practical application, connecting resistance (pads connection of two FPCs), insulation resistance (spacing between pads when two FPCs are connected), and adhesive force of the bonded circuits need to be manipulated to achieve stable connections, herein, the influence of temperature and pressure on the connections was studied. A pressure range from 1.76 MPa to 2.77 MPa and a temperature range from 100 °C to 220 °C, which are popular practical conditions used for bonding in industry, were chosen. The electric resistance among the interconnected circuits (Figure 4a) and insulation resistance were measured, respectively. When the bonding temperature reaches 120 °C, small resistance below 2 Ω was observed. When the temperature reaches 160 °C, very small resistance of 0.1 Ω was observed. Higher pressure can also result in a small resistance. This is a result of the better flow behavior of the adhesive film under higher temperatures and pressures. Although high temperatures and pressures promote tighter bonding between circuits and conducting particles, high temperatures (such as higher than 160 °C) or pressures (such as 2.01 MPa or higher) enhance the flow-ability of the adhesive, causing particles to move from their original positions and become disordered (Figure S10). At these conductions, for example, when the bonding temperature was set at 220 °C, fewer particles can stand between two circuits, thus the conductive resistance obtained increased.

In ACFs interconnections, a higher insulating resistance is preferred in order to avoid affecting circuit operations. The insulation resistance remained above 200 MΩ, showing the high resistance of the adhesives and indicating the conductivity is formed only by the conductive channels of metallic particles.

On the other hand, when bonding temperature or pressure is low (for example, at 100 °C and at a pressure of 1.76 MPa), although the conducting particles’ alignment is very good, the connection among circuit–particles–circuit might not be tight enough. This results in a higher electric resistance. Based on the observed changes in resistance and particle dispersion (Figure S10), a mild bonding temperature range of 140 °C to 160 °C, along with a bonding pressure range of 2.01 MPa to 2.26 MPa, were determined to be suitable parameters for the A-ACFs.

Long term stability was also tested under 85 °C and 85% relative humidity (RH). There is not much change in the resistance of the two connected FPCs bonded under a pressure of 2.01 MPa and temperature of 160 °C, showing the good stability of the bonding by using the A-ACFs (Figure 4b).

### 3.3. Peel Force of the Bonded FPCs Using A-ACFs

Good adhesion, which can be reflected from the peel force, keeps the connected circuits stable when external forces are involved. Figure 5 shows the peel force of two FPC-200s bonded under different temperatures and pressures. The bonding pressure had little effect on the adhesion strength, with only a slight increase in adhesion strength as the pressure increased under the bonding temperatures between 100 °C and 120 °C. This is because at relatively low temperatures, the contact between the adhesive resin in A-ACFs and the FPC mainly depends on the pressure. As the temperature rises (for example at 140 °C or higher), the resin flows more readily, improving contact between the resin and the FPC. This results in relatively high adhesion at high temperatures. Therefore, good adhesive performance (>15 N/cm) can be achieved by using a bonding temperature above 140 °C.

### 3.4. Particles’ Morphologies In-Between the Connected Circuits of the Two Bonded FPCs

**Figure 8:**
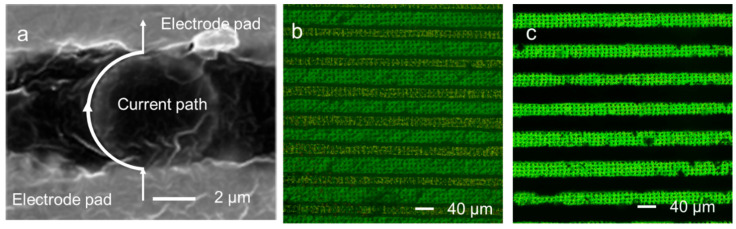
(**a**) Morphologies of conductive particles at 160 °C and 2.01 MPa hot pressing; (**b**) A-ACFs were attached to the FPC-40 surface; (**c**) A-ACFs were bonded between two pieces of FPC-40.

The conductive particles, serving as bridges for the circuit connection, should maintain good contact with both FPCs and without being crushed after bonding. Cross-sectional analyses of the connected pads and particles under different pressures and temperatures were carried out. More detailed and intuitive changes in particle’s morphologies with pressure and temperature were given in Figure S11. At a low bonding temperature of 100 °C and pressure of 1.76 MPa, there are a lot of adhesives between the two pads, showing the particles did not bond strongly between the top pad and bottom pad. At this stage, the resistance between the two pads over 10 Ω (Figure 4a). High bonding temperature (220 °C) and pressure (2.77 MPa) can cause severe particle fractures. At this stage, the particles have poor conductivity due to the destruction of the metal conductive structure on the outer layer of particles. Therefore, at the appropriate bonding temperature (160 °C) and pressure (2.01 MPa), the particles are tightly adhered to the top pad and bottom pad without being broken, providing good electrical conductivity (Figure 8a).

Based on resistance, particle dispersion, peel force, and particle state after bonding, a bonding temperature of 160 °C and pressure of 2.01 MPa optimally achieve low connecting resistance (The average resistance value is 0.10 Ω/0.4 cm^2^, with a standard deviation of 0.0229), orderly particle arrangement, well peel force, and good contacting states between particles and pads.

### 3.5. The Use of the A-ACFs for High-Precision Circuit Bonding

As discussed above the A-ACFs are good interconnection materials for FPC-200. For demonstrating their application, we further use these A-ACFs to bond FPCs with narrower line circuits with a spacing of 40 μm (FPC-40). Using the above optimized bonding condition (160 °C, 2.01 MPa), the connecting resistance and insulation resistance of the two bonded FPC-40s were measured and given in Table S2. The connecting resistance between the two circuits was very small (0.08 Ω) while the insulation resistance remained higher than 200 MΩ (the measurement is given in detail in Figure S1), showing the two interconnected circuits had good connections, while the non-connected circuits has huge electric resistance without electric short circuit, which is required for high-precision connections. Figure 8b,c showed the particles’ distributions on two bonded FPC-40s. Periodic particle alignment was retained for the bonded FPC-40s, which are similar as those of the two bonded FPC-200s.

### 3.6. Performance of the Bonded FPCs Using T-ACFs and the Comparison of A-ACFs with T-ACFs

Figure S12 shows optical micrographs of traditional ACFs (T-ACFs) made with 0.5 wt.% particle contents. The particles are randomly distributed in the T-ACFs. At certain small areas, the particle density varies from dense to sparse, which may result in different particle numbers captured by the solder pads after hot pressing, and excessive particles at the spacing area, leading to inconsistent conduction resistance or circuits short-cut. A-ACFs particle density around 7400 pcs/mm^2^ is familiar with T-ACFs. Using the same bonding conditions (160 °C, 2.01 MPa), Figure S13 shows the 10 circuits lines connecting resistance (using Figure S2 test method) of the two bonded FPC-200s using T-ACFs is higher than those of using A-ACFs bonding, with larger fluctuations across ten measured circuit lines. This difference arises from the uneven dispersion of the conducting particles in T-ACFs, resulting in inconsistent conductive particles across each line and thus greater variation in electric resistance across bonded lines using the T-ACFs. The ordered arrangement of conducting particles in the A-ACFs ensures consistent resistance across lines in the bonded FPC-200s. Although the T-ACFs work well for the bonding of FPC-200 (Table S2), some problems were encountered when the T-ACFs were used for the connection among two FPC-40s.

Although A-ACFs and T-ACFs show little difference in peel strength and FPC-200 performance, the insulation resistance of two bonded FPC-40s using T-ACFs is as low as 0.25 Ω (Table S2). This is extremely low insulation that can greatly harm circuits, indicating electric short-cut can easily occur for high precision circuit connection with narrow spacing. In contrast, the insulation resistance using the A-ACFs still remains stably above 200 MΩ. Therefore, these differences show the obvious advantages of A-ACFs for high bonding precision and circuit stability as compared to T-ACFs.

### 3.7. Flexible Stability of the Bonded FPCs Using A-ACFs

Since the A-ACFs were prepared using PU as adhesives, they not only have excellent conductive properties for bonding, but also possess super high ductility. As shown in Figure S14, during uniaxial stretching with a strain up to 270%, the adhesion and elasticity of the A-ACFs without PET substrate still kept the conducting particles in a relatively stable positional state. This characteristic provides a solid foundation for A-ACFs to bond flexible devices. Thus, we bonded two FPC-200s to test the stability of flexible circuits during operation flexible by A-ACFs (Figure S3). It was found that the flexible circuits exhibited stable performance under bending (0.5 cm bending radius) and 180° twisting test, which was demonstrated by the observation of the constant voltage of 1.5 V and the current at around 15 mA without many fluctuations. This high circuit stability demonstrates that A-ACF joints perform well in flexible circuit applications.

## 4. Conclusions

In this work, we successfully prepared A-ACFs by optimizing the parameters of hot press temperature, time and pressure using a template with microcavity. Regular particles’ arrangements in these A-ACFs were achieved using simple rubbing approach and less than five iterative repeating cycles. A template with a depth of 4 μm is suitable for achieving high transfer efficiency for our designed A-ACFs. These A-ACFs with well-ordered particles’ alignments were used to bond two FPCs to demonstrate their applications. Through analyzing the bonding conditions including temperature, pressure, particle dispersion, and contact state between particles and pad, optimal bonding temperature and pressure were determined to be 160 °C and 2.01 MPa, respectively. The resulting bonded FPCs using A-ACFs not only exhibited stable low resistance (0.1 Ω in 0.4 mm^2^) and high peel force (17.6 N/cm), but also showed stable connections after aging at 85 °C and 85% relative humidity condition for 200 h. This A-ACF can be used not only for bonding FPCs with 200 μm spacing but also for bonding FPCs with 40 μm spacing, enabling higher bonding precision (40 μm) than those using T-ACFs. The two FPC-200s connected using A-ACFs could maintain good circuit stability after 3500 bends and twists, indicating the potential application of the A-ACFs for flexible devices. This work has several significant advantages including simple preparation without using high temperature, high pressure, or complex processes. It is expected that this study may provide valuable insights into improving interconnection precision using the A-ACFs, which will greatly benefit bonding of high-density and high-precision devices.

## Data Availability

The original contributions presented in this study are included in the article/Supplementary Material. Further inquiries can be directed to the corresponding authors.

## Supplementary Materials

The following supporting information can be downloaded at: https://www.mdpi.com/article/10.3390/ma18214927/s1. Figure S1: Connecting resistance and insulation resistance test method for FPC-40; Figure S2: Connecting resistance and insulation resistance test method for FPC-200; Figure S3: Flexible stability testing of flexible circuits bonded by A-ACFs; Figure S4: Effect of Adhesion Energy on Particle Filling Process; Figure S5: Effect of Adhesion Energy on Particle Transfer Process; Figure S6: SEM of particles transferred on PU films from the template with 4 m depth under different temperatures; Figure S7: SEM of particles transferred on PU films from the template with 5 μm depth under different temperatures; Figure S8: Time dependent particle transfer efficiency; Figure S9: Particle transfer efficiency at different pressures; Figure S10: Effect of pressure and temperature on particles dispersion; Figure S11: Effect of temperatures and pressures on the morphologies of particles; Figure S12: Optical microscope image of T-ACFs attached to FPC-40; Figure S13: Comparison of resistance of A-ACFs with T-ACFs; Figure S14: Flexible display of A-ACFs; Table S1: The effect of spin coating cycles on thickness; Table S2: Comparison of resistance in Circuits with Different Spacing using A-ACFs and T-ACFs.
