# Supplementary material for: Array-Patterned Anisotropic Conductive Films for High Precision Circuit Interconnection
Source: Materials (Basel). 2025 Oct 28;18(21):4927. doi: 10.3390/ma18214927 (PMC12610642; doi:10.3390/ma18214927)
Supplement: Supplementary file 1 [file materials-18-04927-s001.zip › materials-3903761-supplementary.pdf]

Supporting Information

Array-patterned anisotropic conductive films for high precision circuit interconnection

Changxiang Hao[a], Junde Chen[a], Yonghao Chen[a], Ge Cao[b,c,*], Xing Cheng[a,*], Yanqing Tian[a,*]

[a] *Department of Materials Science and Engineering, Southern University of Science and Technology, Shenzhen 518055, People's Republic of China*

b Institute of Corrosion Science and Technology, Guangzhou, 510530, China
c School of Textile, Guangdong Polytechnic, Gaoming, Foshan, 528000, China
** Corresponding authors E-mail addresses: gcao@icost.ac.cn(Ge Cao); chengx@sustech.edu.cn (X. Cheng); tianyq@sustech.edu.cn (Y. Tian).*

* Corresponding authors

Email addresses: chengx@sustech.edu.cn (X. Cheng), tianyq@sustech.edu.cn (Y. Tian).

Table of Contents

Figure S1. Connecting resistance and insulation resistance test method for FPC-40…...3

Figure S2. Connecting resistance and insulation resistance test method for FPC-200…4

Figure S3. Flexible stability testing of flexible circuits bonded by A-ACFs. ..………..5

Figure S4. Effect of Adhesion Energy on Particle Filling Process. … 6

Figure S5. Effect of Adhesion Energy on Particle Transfer Process. . … . . . . … … 7

Figure S6. SEM of particles transferred on PU films from the template with 4 μm depth under different temperatures. …..……………………………………..…....8

Figure S7. SEM of particles transferred on PU films from the template with 5 μm depth under different temperatures. ………………..………..................………..9

Figure S8. Time dependent particle transfer efficiency. ……………………..……..10

Figure S9. Particle transfer efficiency at different pressures. ………..………..……11

Figure S10. Effect of pressure and temperature on particles dispersion. …………..……12

Figure S11. Effect of temperatures and pressures on the morphologies of particles. ...13

Figure S12. Optical microscope image of T-ACFs attached to FPC-40. ……………..…14

Figure S13. Comparison of resistance of A-ACFs with T-ACFs. ……….……………15

Figure S14. Flexible display of A-ACFs. ……………………….……………………..16

Table S1. The effect of spin coating cycles on thickness. ……………...……………17

Table S2. Comparison of resistance in Circuits with Different Spacing using A-ACFs and T-ACFs. …………………………………………………………18

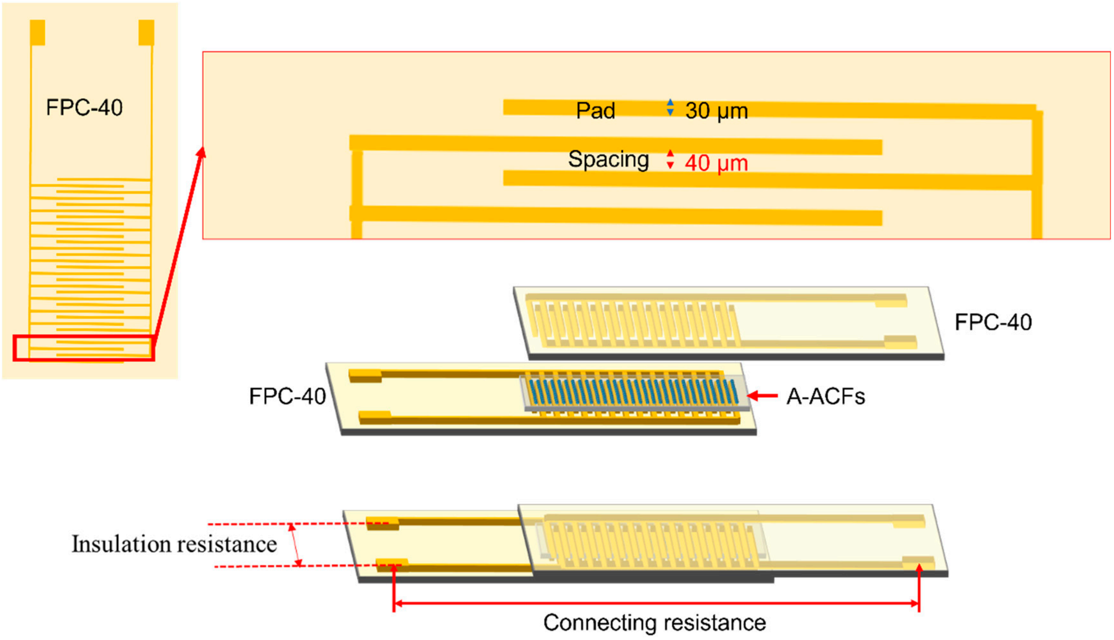

Figure S1. Insulation resistance and connecting resistance test method for FPC-40.

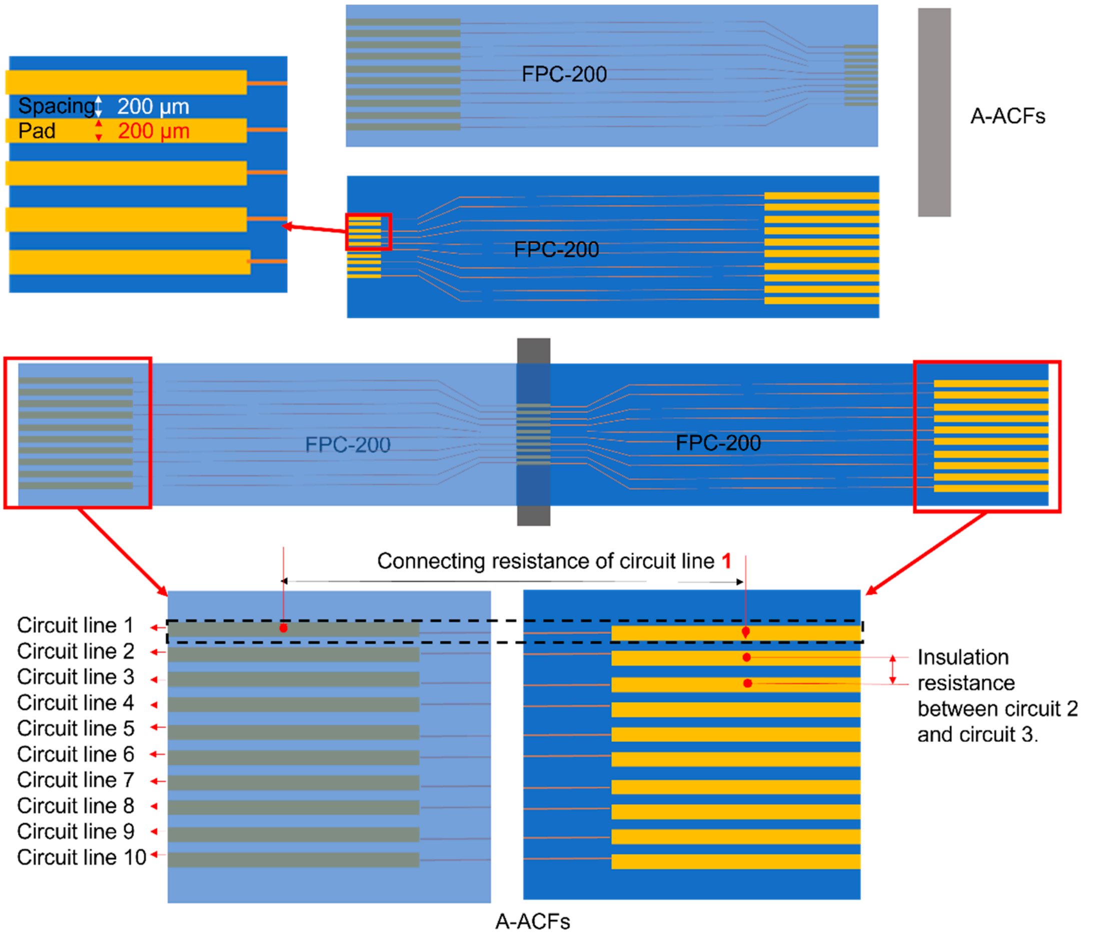

Figure S2. Test method of the connecting resistance and insulation resistance for FPC-200.

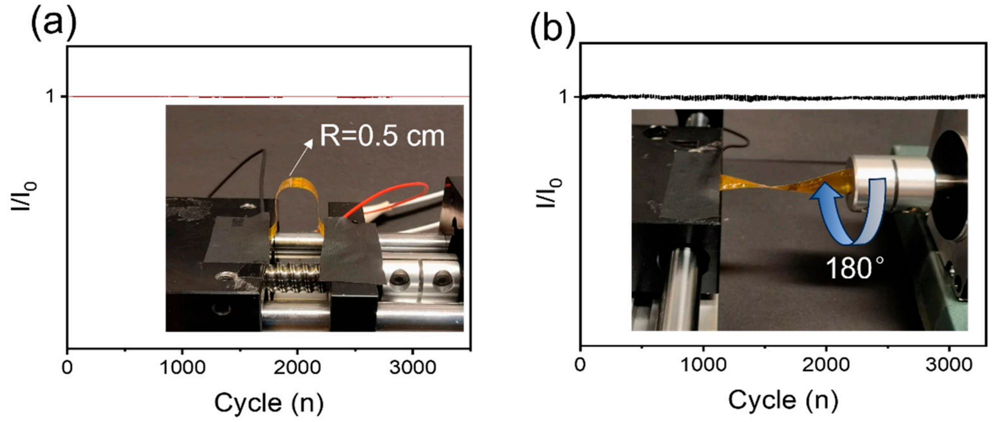

Figure S3. Flexible stability testing of flexible circuits bonded by A-ACFs. (a) Bending test; (b) Twisting test.

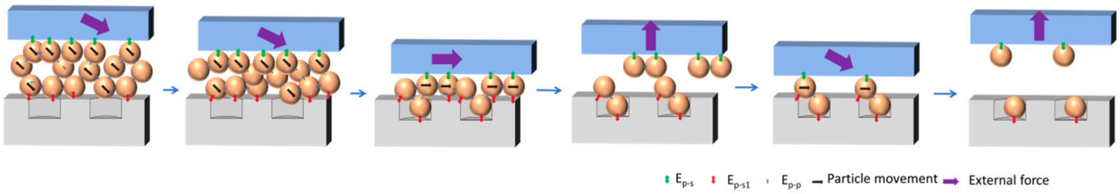

Figure S4. Schematic Diagram of the Effect of Adhesion Energy on Conductive Particles During the Conductive Particle Filling Process.

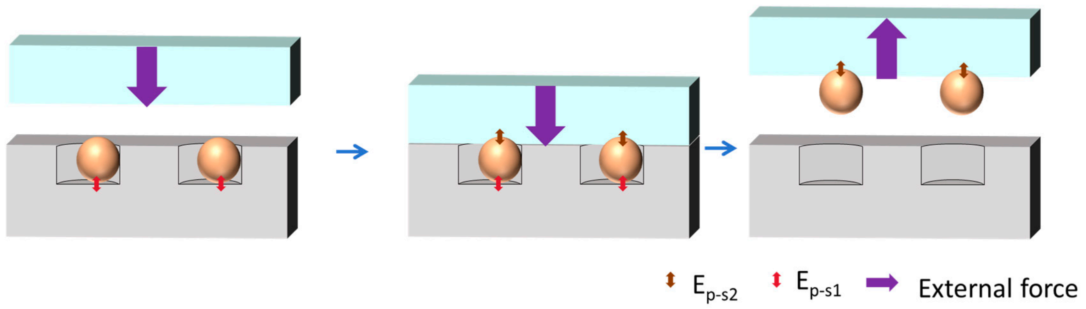

FigureS5. Schematic Diagram of the Effect of Adhesion Energy on Conductive Particles During the Conductive Particle Transfer Process.

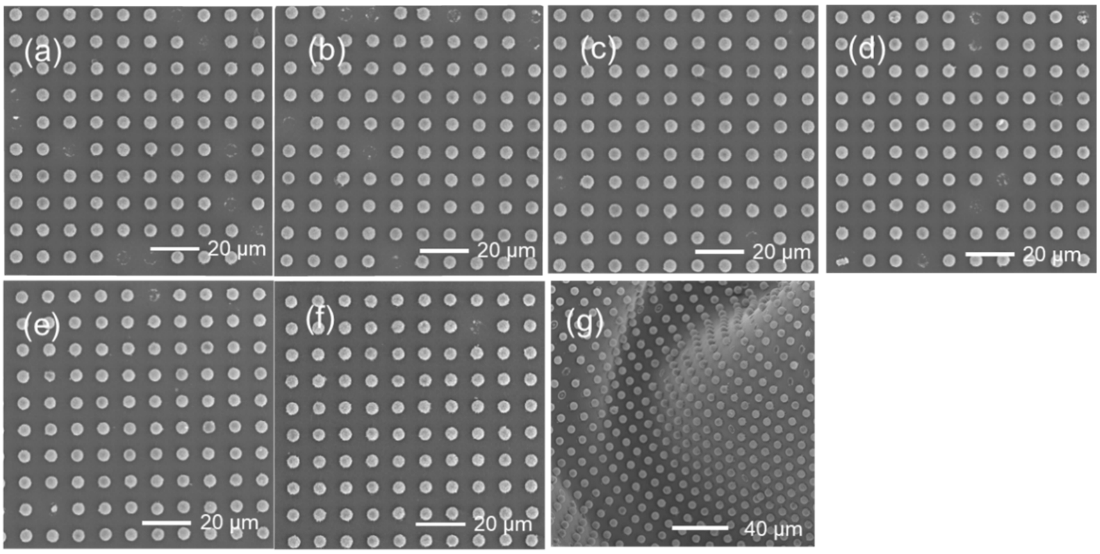

Figure S6. SEM of particles transferred on PU films from the template of 5 times particles filling cycles at 3.76 KPa for 10 s using the microcavity with 4 μm depth under different temperatures. (a) 25°C; (b) 40°C; (c) 60°C; (d) 80°C; (e) 100°C; (f) 120°C; and (g) 140°C.

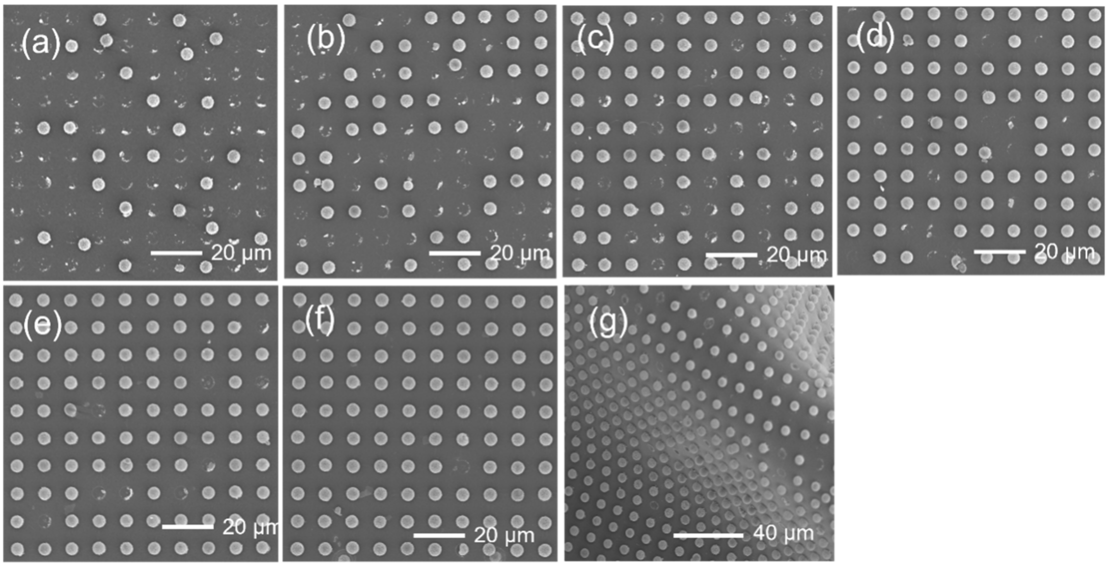

Figure S7. SEM of particles transferred on PU films from the template of 5 times particles filling cycles at 3.76 KPa for 10 s using the microcavity with 5 μm depth under different temperatures. (a) 25°C; (b) 40°C; (c) 60°C; (d) 80°C; (e) 100°C; (f) 120°C; (g) 140°C.

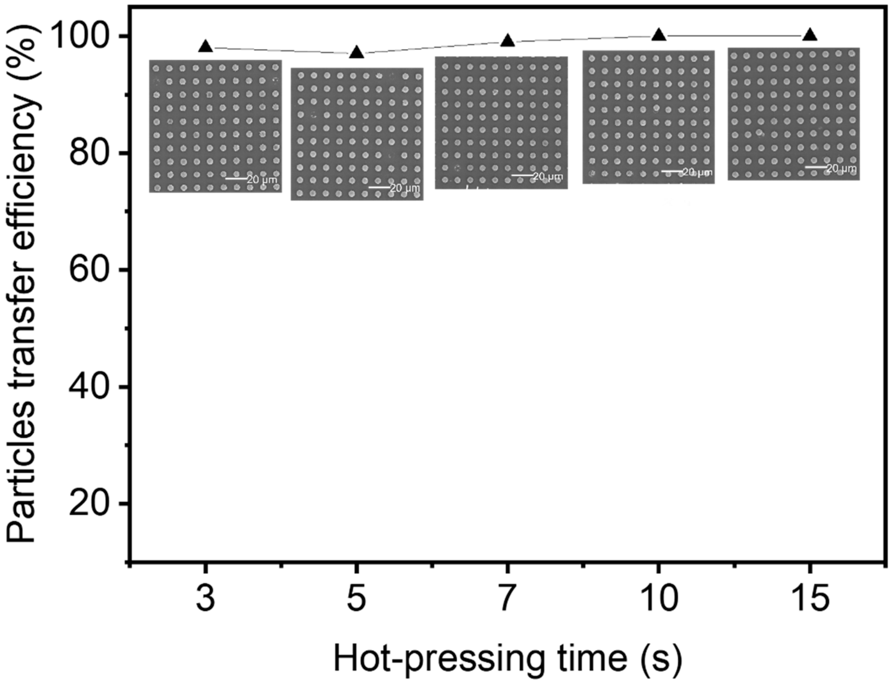

Figure S8. Time dependent particle transfer efficiency under 3.76 KPa and at 120 C for the 4 μm depth microcavity. (a) 3 s; (b) 5 s; (c) 7 s; (d) 10 s; (e) 15 s.

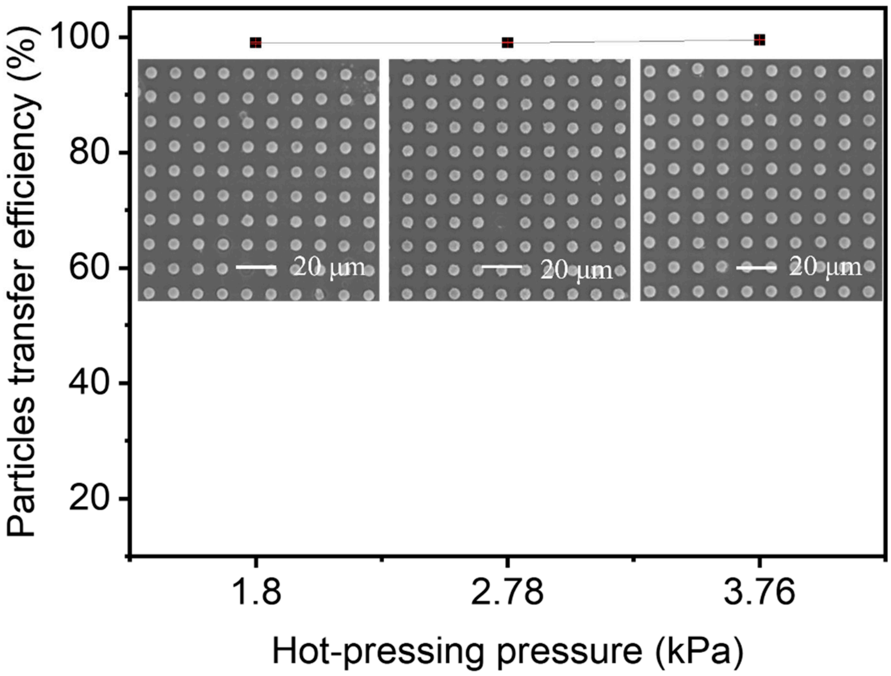

Figure S9. Particle transfer efficiency at different pressures at 120°C, microcavity depth is 4 μm, for 10 s.

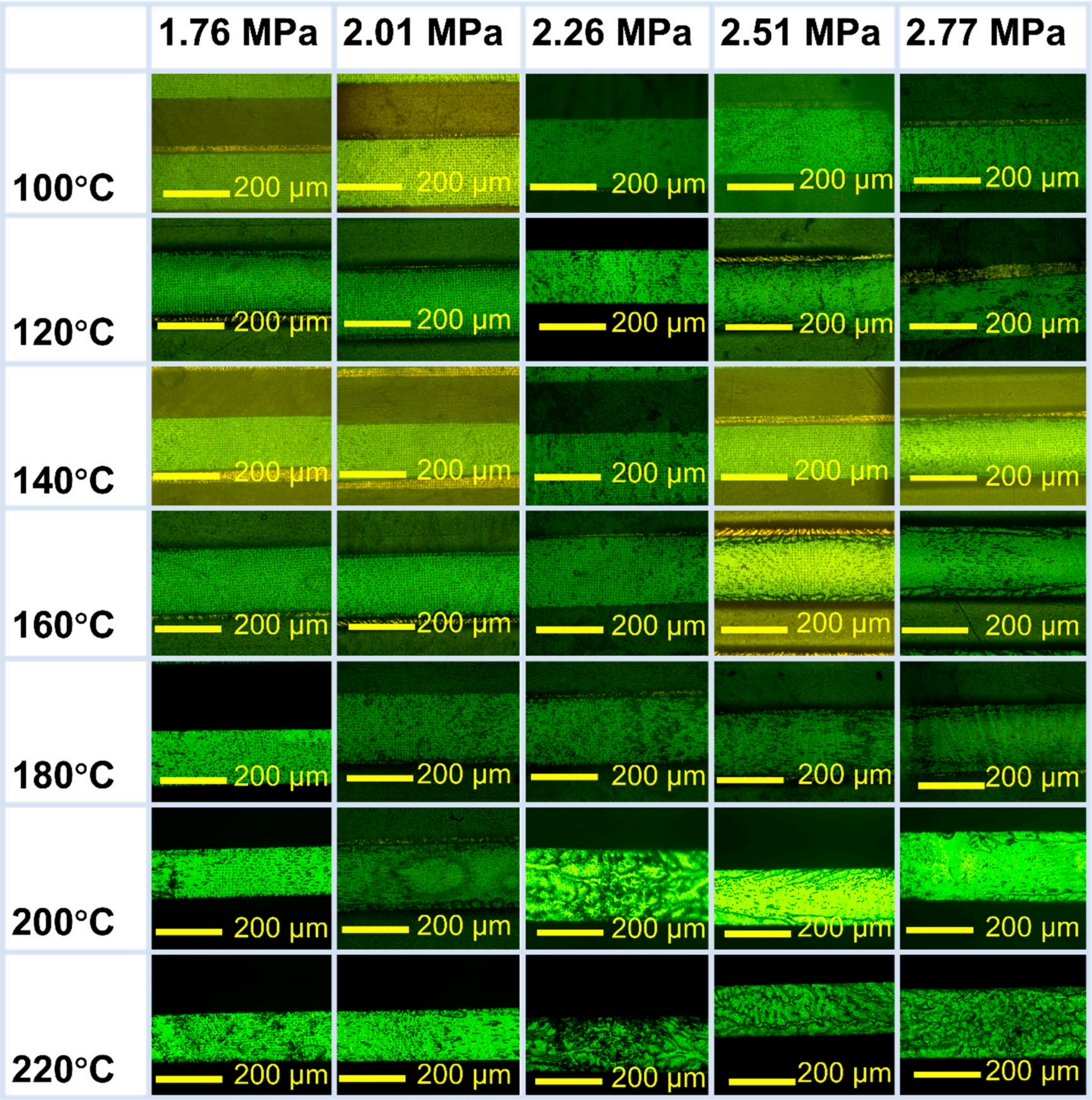

Figure S10. Effect of pressure (lengthways) and temperature (transverse) on particles dispersion in bonded FPC-200 circuits.

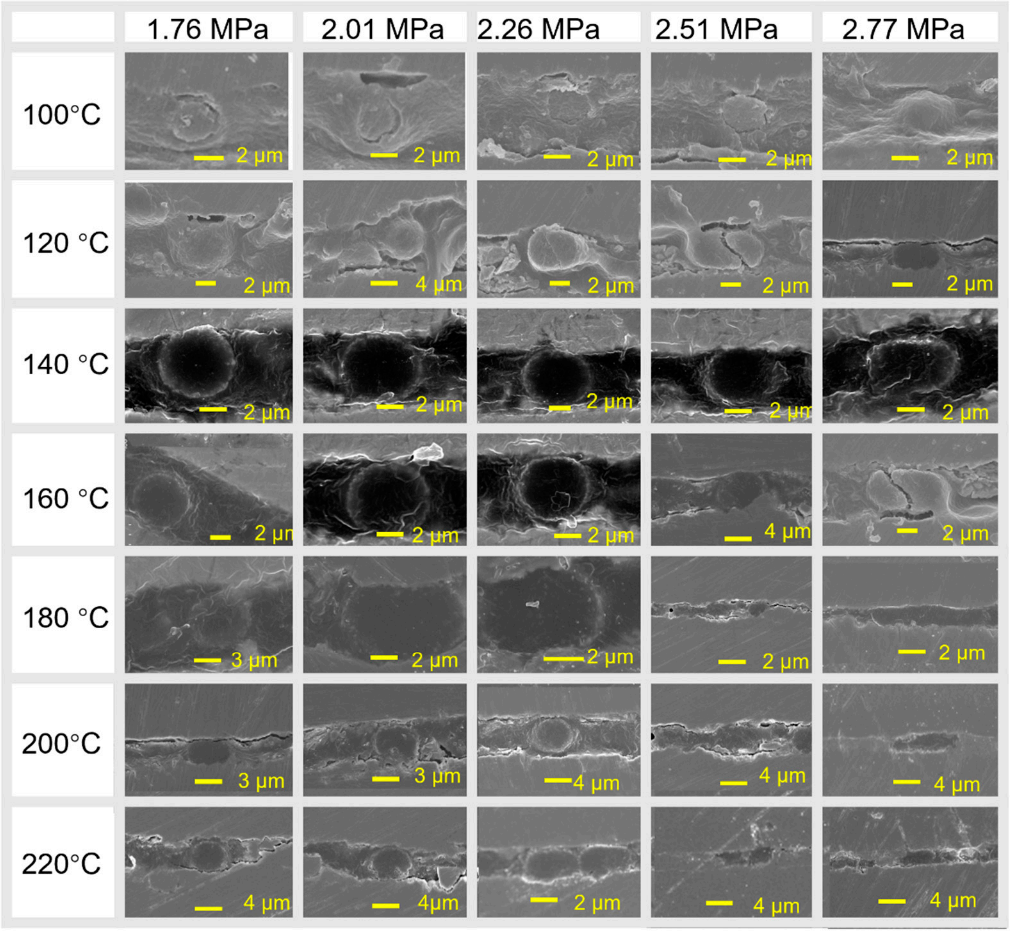

Figure S11. Effect of temperatures (transverse) and pressures (lengthways) on the morphologies of the conductive particles in bonded circuit of FPC-200.

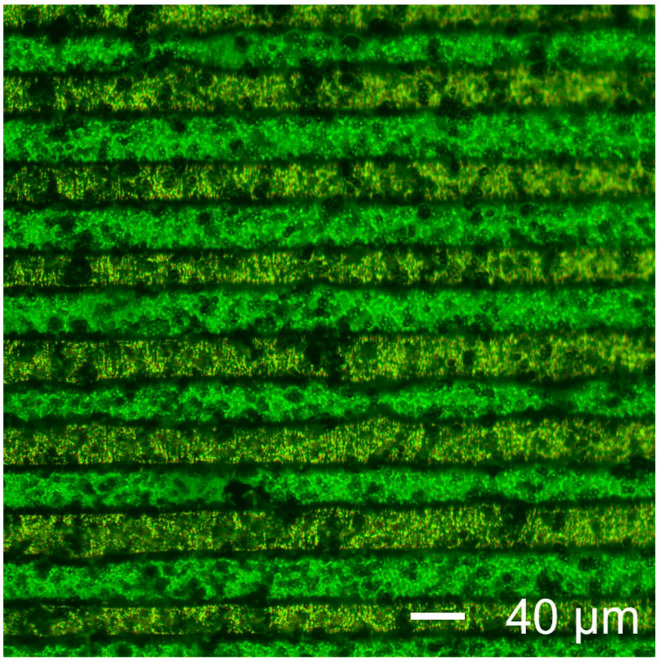

Figure S12. Optical microscope image of T-ACFs attached to FPC-40.

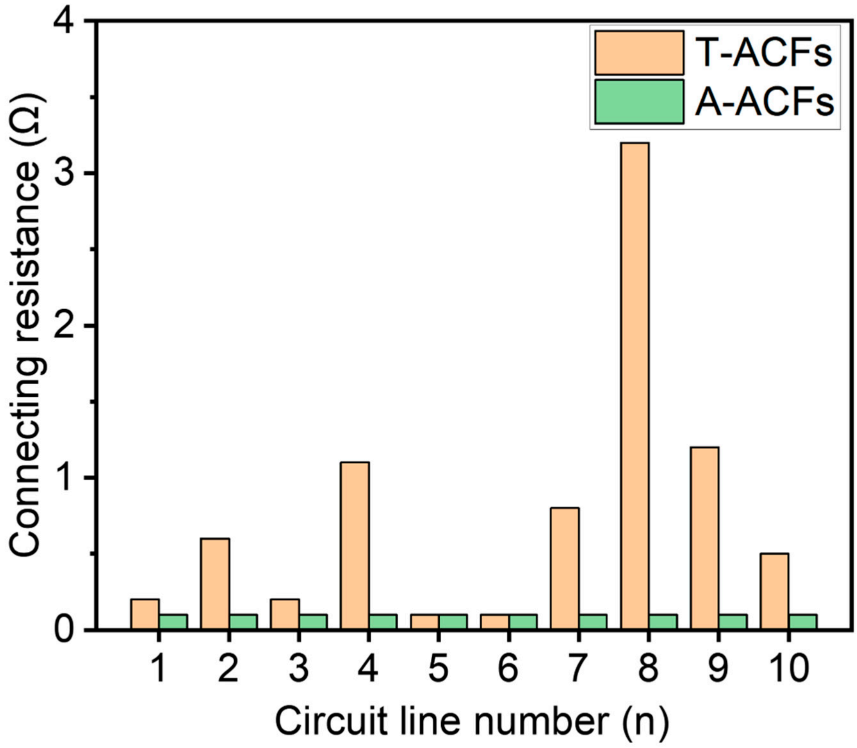

Figure S13. Comparison of connecting resistance between A-ACFs and T-ACFs after bonded FPC-200.

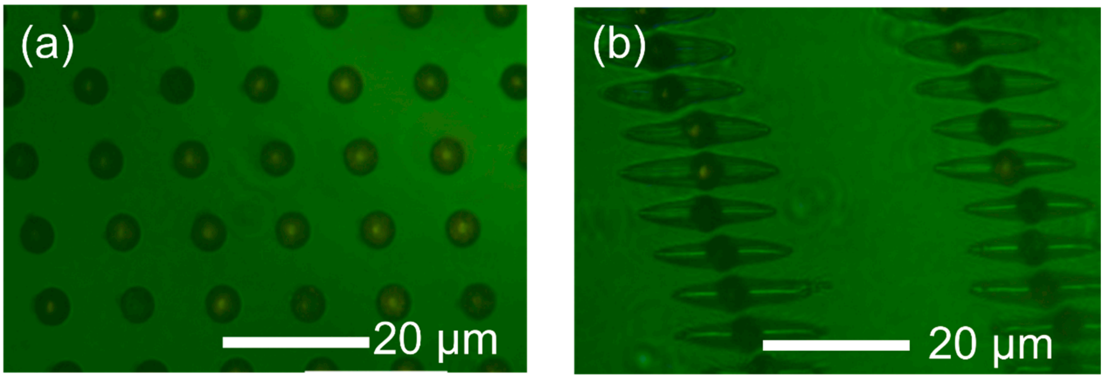

Figure S14. Flexible display and stability testing of flexible circuits bonded by A-ACFs. (a) A-ACFs before stretching; (b) Stretch 270% of A-ACFs.

Table S1. The effect of spin coating and drying cycles on thickness.

**Cycles**<br>**(Low speed: 450r/min,10s**<br>**High speed: 5000r/min,20s)**	1	2	3	4	5	6	7
**Thickness (μm)**	3	7	10	14	19	25	34

Table S2. Resistance variations of different bonded FPCs using A-ACFs or T-ACFs.

	FPC-40 connecting resistance	FPC-40 insulation resistance	FPC-200 connecting resistance	FPC-200 insulation resistance	Peel force
**A-ACFs**	0.08 Ω	＞200 MΩ	0.1 Ω	＞200 MΩ	17.1 N/cm
**T-ACFs**	0.07 Ω	0.25Ω	0.1 Ω	＞200 MΩ	16.8 N/cm
